# HLA class II DR and DQ genotypes and haplotypes associated with rheumatic fever among a clinically homogeneous patient population of Latvian children

**DOI:** 10.1186/ar2216

**Published:** 2007-06-10

**Authors:** Valda Stanevicha, Jelena Eglite, Dace Zavadska, Arturs Sochnevs, Ruta Shantere, Dace Gardovska

**Affiliations:** 1 Department of Pediatrics, Riga Stradins University, Vienîbas gatve 45, Riga, LV1004, Latvia; 2 Department of Imunology, Riga Stradins University, Dzirciema iela 16, Riga, LV1007, Latvia; 3 Children Clinical University Hospital, Vienîbas gatve 45, Riga, LV1004, Latvia

## Abstract

The HLA system is being paid more and more attention because it is very significant in polymorphous immunological reactions. Several studies have suggested that genetic susceptibility to rheumatic fever (RF) and rheumatic heart disease (RHD) is linked to HLA class II alleles. We hypothesized that HLA class II associations within RHD may be more consistent if analysed amongst patients with a relatively homogeneous clinical outcome. A total of 70 RF patients under the age of 18 years were surveyed and analysed in Latvia. HLA genotyping of DQA1, DQB1 and DRB1 was performed using PCR with amplification with sequence-specific primers. We also used results from a previous study of DQB1 and DRB1 genotyping. In the RF patients, HLA class II DQA1*0401 was found more frequently compared to DQA1*0102. In the RF homogeneous patient groups, DQA1*0402 has the highest odds ratio. This is also the case in the multivalvular lesion (MVL) group, together with DQA1*0501 and DQA1*0301. In the chorea minor patients, DQA1*0201 was often found. Significant HLA DQA1 protective genotypes were not detected, although DQA1 genotypes *0103/*0201 and *0301/*0501 were found significantly and frequently. In the distribution of HLA DRB1/DQA1 genotypes, *07/*0201 and *01/*0501 were frequently detected; these also occurred significantly often in the MVL group. The genotype *07/*0201 was frequently found in Sydenhamn's chorea patients that had also acquired RHD, but DRB1*04/DQA1*0401 was often apparent in RF patients without RHD. In the distribution of HLA DQA1/DQB1 genotypes, both in RF patients and in the homogeneous patient groups, the least frequent were *0102/*0602-8. The genotype DQA1*0501 with the DQB1 risk allele *0301 was often found in the MVL group. The genotype *0301/*0401-2 was frequently found in the RF and Sydenhamn's chorea patient groups. The haplotype *07-*0201-*0302 was frequently found in RF and homogeneous patient groups, including the MVL group. In addition, haplotypes *04-*0401-*0301 and *04-*0301-*0401-2 were frequent amongst patients with Sydenhamn's chorea. The protective alleles DQA1*0102 and DQB1*0602-8 in the haplotype DRB1*15 were less frequently found in RF patients. The results of the present study support our hypothesis and indicate that certain HLA class II haplotypes are associated with risk for or protection against RHD and that these associations are more evident in patients in clinically homogeneous groups.

## Introduction

The HLA system is being paid more and more attention because it is very significant in polymorphous immunological reactions. The role of genetic factors in the pathogenesis of rheumatic fever (RF), an autoimmune disease, was documented many decades ago. As a result, investigative efforts were focused on the genetic markers of susceptibility to this preventable disease.

RF is an autoimmune sequela of group A streptococcal infections and one of the leading causes of morbidity and mortality in many parts of the world. The disease is often preceded by RF episodes that may, in susceptible individuals, progress to a chronic valvular disease. The relatively low occurrence of RF after untreated streptococcal tonsillopharyngitis (in 0.3% to 3% of patients) suggests the involvement of host genetic factors in the susceptibility to RF with consequential progression to rheumatic heart disease (RHD).

The basis of the autoimmune processes that contribute to the development of RHD is T-cell molecular mimicry between streptococcal and heart proteins. RHD is initiated by certain serotypes connected with group A streptococcus M protein. Guilherme and colleagues [1-4] suggest that peptides M1 and M5 cause RHD, and that peptides M6 and M24 cause brain damage in patients who have the HLA DRB1*07/53 genotype combined with severe RHD.

Several studies have suggested that genetic susceptibility to RF and RHD is linked to HLA class II alleles [5-8]. However, there have been apparent discrepancies as to the nature of the susceptibility and/or protective alleles. Several years ago these discrepancies could have been associated with different types of laboratory methods, but they may also be partly because of ethnic differences in the distribution of HLA alleles and the contributions of other genes that may have been displayed amongst different populations. A distinct linkage disequilibrium occurs with HLA DR or DQ alleles.

Antimicrobial peptides are released at epithelial surfaces and disrupt the membranes of many microbial pathogens. Toll-like receptors on epithelial cells and leukocytes recognize a range of microbial molecular patterns and generate intracellular signals for activation of a range of host responses [3,4,9]. These innate immune mechanisms and their interactions in the defence against infection provide the host with the time needed to mobilize the slower developing mechanisms of adaptive immunity, which might protect against subsequent challenges [10,11].

Genetic associations are more likely to be detected in clinically homogeneous groups of patients, and it is thus important to separate carditis patients from patients with different RF sequelae. However, ethnic differences also play a role [12-22]. We noted that in studies in which RF patients with carditis were analyzed separately from those with other RF sequelae, or in which only RHD patients, the majority of whom had mitral valve regurgitation (MVR), were studied, the reported HLA associations were rather similar [18]. Based on these observations, we hypothesized that HLA class II associations with RHD may be more consistent if analyzed in patients with a relatively homogeneous clinical outcome.

During the 1980s and 1990s, the Latvian morbidity rate from RF increased rapidly, reaching a peak of incidence of 11 per 100,000 children. Currently, the incidence is 0.1 per 100,000 children. Since then the course of RF has also changed; currently, the inflammation of cardiac valves is accompanied by Sydenham's chorea, but without laboratory activity of inflammation. Also, during the course of RF, 67.1% of the patients acquired RHD, which is higher than the rate in other countries; for example, in Brazil, 30% of patients acquired it during this time period [12,20,21]. This directed us to survey all children born in the years 1984 to 2002 that had contracted RF. We studied HLA class II DQB and DRB alleles in homogeneous patient groups using PCR.

The results of a previous study [23] on HLA class II DQ and DR alleles in homogeneous patient groups support our hypothesis and indicate that the HLA genotype DRB1*0701, DQB1*0302, DQB1*0401-2 is statistically significant as a predisposing factor for RF, but that the genotype DRB1*06-DQB1*0602-8 is possibly associated with protection against RF and the development of RHD.

Our data indicate that certain class II alleles/genotypes are associated with risk for or protection against RF and RHD and these associations appear to be stronger and more consistent when analysed in patients with relatively more homogeneous clinical manifestations.

Recently, we have also studied DQA alleles in these homogeneous RF patient groups and have determined HLA class II DQA, DQB, DRB genotypes and haplotypes. We compare the results of our studies to those from studies in other countries with typically high incidence rates of RF and RHD in order to maintain the hypothesis concerning the necessity of conducting genetic research into homogeneous patient groups and to prove the significance of ethnic differences.

## Materials and methods

### Subjects

This study includes 70 white children – 48 boys (68.5%) and 22 girls (31.4%) – in Latvia under the age of 18 who had RF during the period 1984 to 2002. There were 23 (32.8%) patients less than 7 years of age and 47 (67.1%) over 7 years of age. The RF diagnosis was confirmed according to the Jones criteria. Eight RF patients had chorea minor. As a result of RF, 47 patients (67.1%) had developed RHD. Cardial valve damage was diagnosed by echocardiography and/or heart catheterisation. RHD patients were further split into groups with mitral valve regurgitation (MVR; *n *= 24 (34.3%)), aortal valve regurgitation (AVR; *n *= 3 (4.3%)) and MVR + AVR or multivalvular lesion (MVL) (*n *= 20 (28.6%)). Only 23 of the patients (32.8%) had fully recovered by the age of 18. A RF set-back was recorded for 15% of the patients because they had not received prolonged penicillin treatment. Data for healthy individuals (*n *= 100) were obtained from the Databank of the Immunology Institute of Latvia. The above individuals were free of autoimmune disease and had no family history of RF. In both groups (RF patients and healthy individuals) HLA class II alleles were determined by PCR.

### DNA isolation

Genomic DNA was extracted from proteinase K-treated peripheral blood leukocytes using the routine salt-off method. The DNA was stored in TE buffer (10 ml Tris-HCl, pH 7.5, 2 ml 0.5 M Na_2 _EDTA/L d-H_2_O). Extracted genomic DNA, which was used for genotyping, was stored at -20°C. The DNA concentration (100 to 200 μg/ml) was determined using the fluorescent method with a DNA fluorimeter [24].

### HLA DR and DQ genotyping by PCR

Low-resolution HLA DR typing for DRB1*01 through 18 and DQ typing for DQA1*0101, *0102, *0103, *0201, *0301, *0401, *0501, *0601 and DQB1*0201-202, *0301-305, *0401-402, *0501-504 and *0601-608 was performed by PCR using amplification with sequence-specific primers (PCR-SSP) [25]. The reaction mixture (15 μl) included 1 μl DNA, 1.5 μl PCR buffer (50 mM KCl, 1.5 mM MgCl_2_, 10 mMTris-Cl, pH 8.3), 0.6 μl dNTPs (25 mmol/l), 1.0 μl specific primers (0.2 mmol/l), and 0.5 U of the Taq DNA polymerase (Promega, Madison, WI, USA). In addition, the internal positive control primer pair, C3 and C5, was included in all reaction mixtures in a five-fold lower concentration than the allele- and group-specific primers.

Each reaction mixture was subjected to 36 amplification cycles consisting of denaturation at 94°C for 60 s one cycle, annealing at 94°C for 20 s and 67°C for 2 s, seven cycles; extension at 93°C for 5 s and 65°C for 4 s, repeat the cycle 28 times.

PCR products were visualized by agarose gel electrophoresis. After addition of the 2 M loading buffer, the PCR reaction mixtures were loaded in agarose gels pre-stained with ethidium bromide (0.5 μk/ml gel). The gels were run for 15 minutes at 10 V/cm in 0.5 mM TBE buffer and then examined under UV illumination and recorded [24-30].

### Statistics

The HLA DRB1, DQA1 and DQB1 allele frequencies in patients and control subjects were compared. Typing of all three loci was performed on all patients and control subjects. Allele and haplotype frequencies of HLA class II were determined by gene counting tests. The differences between predisposing to and protecting against RHD were measured using the odds ratio (OR) method: OR = ad/bc or OR = (2a + 1)(2d + 1)/(2d + 1)(2c + 1) when b or c = 0. The statistical significance was examined by Fisher's exact test in RHD and the subgroup of RHD. Allele frequencies (AF) were calculated using the following formula: AF(%) = the sum of the allele/2n × 100, where n is the sum of the total number of individuals analysed. Haplotype frequencies (HF) were determined by the method of gene counting and calculated using the formula: HF(%) = sum of given haplotype/2n × 100. *P *values were calculated using Epi Info software version 6 [31] with 95% confidence intervals, and Mantel-Hanzszel and Fisher exact correction for small numbers [32].

## Results

### Distribution of HLA DQA1 alleles and genotypes in RF patients

HLA class II DQA1*0401 (OR = 3.31, *p *< 0.01) was found more frequently in RF patients than in the control group, while the DQA1*0102 (OR = 0.34, *p *< 0.001) was found less frequently in RF patients than in the control group (Table 1).

**Table 1:** The frequency of DQA1* alleles in rheumatic fever patients and healthy controls

DQA1* alleles	RF (*n *= 140)	Percent	Controls (*n *= 200)	Percent	Odds ratio	*P *value
*0101	18	13	29	14	0.87	<0.66
*0102	12	9	43	21	0.34	<0.001
*0103	15	11	16	8	1.38	<0.39
*0201	17	12	24	12	1.01	<0.97
*0301	22	16	27	13	1.19	<0.57
*0401	15	11	7	3	3.31	<0.01
*0501	41	29	48	24	1.31	<0.27
*0601	0	-	6	3	ND	-

ND, not determined; RF, rheumatic fever.

In the RF homogeneous patient groups (Table 2), DQA1*0401 has the highest OR. This is also the case in the MVL group, together with DQA1*0501 (OR = 3.25, *p *< 0.03) and DQA1*0301 (OR = 3.45, *p *< 0.02). In Sydenham's chorea patients, DQA1*0201 was often found (OR = 3.33, *p *< 0.05)

**Table 2:** DQA1 allele distribution in rheumatic heart disease patients compared with control subjects

	Allele				
Group	DQA1*0102	DQA1*0201	DQA1*0401	DQA1*0501	DQA1*0301

All RF (*n *= 140)	0.13/0.34 (0.001)	0.12	0.11/3.31 (0.01)	0.29	0.16
MVR (*n *= 48)	0.08	0.08	0.08	0.29	0.23
MVL (*n *= 40)	0.23	0.10	0.07/4.87 (0.05)	0.37/3.25 (0.03)	0.17/3.45 (0.02)
Chorea minor (*n *= 16)	0.13	0.31/3.33 (0.05)	0.00	0.19	0.31/2.91 (0.05)
Without RHD (*n *= 46)	0.13	0.17	0.06	0.24	0.13
Control subjects (*n *= 200)	0.21	0.12	0.03	0.24	0.13

Values are allele frequency/odds ratio (*p *value) and are reported only for significant associations (*p *< 0.05); *n *= number of haplotypes (for example, 140 alleles from 70 individuals and 200 alleles from 100 individuals). MVL, multivavular lesion (mitral regurgitation + aortic valve regurgitation; MVR, mitral valve regurgitation; RF, rheumatic fever; RHD, rheumatic heart disease.

The DQA1*0102 allele was absent in all RF patients, whereas its frequency was 9% in control subjects (*p *< 0.001), but it showed no significant protective effect in the homogeneous patient groups.

Significant HLA DQA1 protective genotypes were not found (Table 3), although DQA1 genotypes *0103/*0201 (OR = 7.62, *p *< 0.03) and *0301/*0501 (OR = 2.61, *p *< 0.009) were found more frequently.

**Table 3:** Significant HLA DQA1 genotypes associated with predisposition/protection in all RF patients

DQA1	RF (*n *= 70)	Percent	Controls (*n *= 100)	Percent	Odds ratio	P value (Fisher)
*0101-*0501	13	18	8	8	2.62	<0.039
*0102/*0201	1	1.5	4	4	0.35	<0.330
*0102/*0501	8	11	13	13	0.86	<0.759
*0103/*0201	**5**	**7**	**1**	**1**	**7.62**	**<0.03**
*0103/*0501	4	6	2	2	2.97	<0.197
*0201/*0301	3	4	5	5	0.85**	<0.829
*0301/*0501	**10**	**14**	**6**	**6**	**2.61**	**<0.009**

Entries in bold are statistically significant associations for patients versus controls. RF, rheumatic fever. **Not significant result

### Distribution of HLA DRB1/DQA1 genotypes

The most frequently found DRB1/DQA1 genotypes in RF patients are *07/*0201 (OR = 2.01, *p *< 0.06) and *01/*0501 (OR = 3.18, *p *< 0.005) (Table 4), which also occur significantly often in the MVL group (OR = 5.69, *p *< 0.001). The genotype *07/*0201 has often been found in Sydenhamn's chorea patients (OR = 3.72, *p *< 0.04), but DRB1*04/DQA1*0401 is found in RF patients without RHD (OR = 11.1, *p *< 0.004).

**Table 4:** DRB1/DQA1 genotype distribution in rheumatic fever and rheumatic heart disease patients compared with control subjects

	Genotype		
Group	*07/*0201	*01/*0501	*04/*0401

All RF (*n *= 70)	0.09/2.01 (0.06)	0.08/3.18 (0.005)	0.01/0.23
MVR (*n *= 24)	0.06	0.06	ND
MVL (*n *= 20)	0.1	0.01/5.69 (0.001)	ND
Sydenham's chorea (*n *= 8)	0.18/3.72 (0.04)	0.05	ND
Without RHD (*n *= 23)	0.13/2.79 (0.02)	0.06	0.001/11.10 (0.004)
Control subjects (*n *= 100)	0.05	1.44/0.23	0.19/0.65

Values are allele frequency/odds ratio (*p *value) and are reported only for significant associations (*p *< 0.05); *n *= number of haplotypes (for example, 140 alleles from 70 individuals and 200 alleles from 100 individuals). MVL, multivalvular lesion (mitral regurgitation + aortic valve regurgitation; MVR, mitral valve regurgitation; ND, not determined; RF, rheumatic fever; RHD, rheumatic heart disease.

### Distribution of HLA DQA1/DQB1 genotypes

Both in the RF patients and the homogeneous patient groups the least frequent DQA1/DQB1 genotypes are *0102/*0602-8 and *0501/*0201-2 (Table 5). The genotype DQA1*0501 with the DQB1 risk allele *0301 was often found in RF patients (OR = 2.10, *p *< 0.01), the MVL group (OR = 3.35, *p *< 0.001) and also in patients without RHD (OR = 2.58, *p *< 0.03). The DQA1/DQB1 genotype *0301/*0402 has been found significantly in RF and Sydenhamn's chorea patient groups, but not in RHD groups.

**Table 5:** DQA1/DQB1 genotype distribution in rheumatic fever and rheumatic heart disease patients compared with control subjects

	Genotype			
Group	*0102/*0602-8	*0501/*0301	*0501/*0201-2	*0301/*0402

All RF (*n *= 70)	0.09	0.19/2.10 (0.01)	0.01/0.26 (0.05)	0.03/7.06 (0.03)
MVR (*n *= 24)	0.06	0.15	0.02	ND
MVL (*n *= 20)	0.12	0.25/3.35 (0.01)	ND	0.02
Sydenham's chorea (*n *= 8)	0.12	0.12	ND	0.12/38.83 (0.005)
Without RHD (*n *= 23)	0.12	0.22/2.58 (0.03)	0.02	0.07/17.48 (0.0005)
Control subjects (*n *= 100)	0.12/198.63 (0.00)	0.11	0.05	0.004

Values are allele frequency/odds ratio (*p *value) and are reported only for significant associations (*p *< 0.05); *n *= number of haplotypes (for example, 140 alleles from 70 individuals and 200 alleles from 100 individuals). MVL, multivavular lesion (mitral regurgitation + aortic valve regurgitation); MVR, mitral valve regurgitation; ND, not determined; RF, rheumatic fever; RHD, rheumatic heart disease.

### Distribution of DRB1-DQA1-DQB1 haplotype

The DRB1-DQA1-DQB1 haplotype *07-*0201-*0302 (OR = 21.94, *p *< 0.001) was frequently found in RF and homogeneous patient groups, including the MVL group (OR = 26.0, *p *< 0.001) and patients without RHD (OR = 35.1, *p *< 0.002) (Table 6). In addition, the DRB1-DQA1-DQB1 haplotypes *04-*0401-*0301 (OR = 16.6, *p *< 0.003) and *04-*0301-*0401-2 (OR = 78.0, *p *< 0.0001) were found frequently amongst patients with Sydenhamn chorea. The protective alleles DQA1*0102 and DQB1*0602-8 in haplotype DRB1*15 showed no significant protective effects in RF patients.

**Table 6:** DRB1-/DQA1-DQB1 haplotype distribution in rheumatic fever and rheumatic heart disease patients compared with control subjects

	Haplotype			
Group	*04-*0401*-0301	*04-*0301-*0401-2	*07-*0201-*0302	*15-*0102-*0602-8

All RF (*n *= 70)	0.01	0.02	**0.04/21.94 (0.001)**	0.08/0.88 (0.67)
MVR (*n *= 48)	0.02	0.02/5.07 (0.25)	0.04/2.27 (0.27)	0.06
MVL (*n *= 40)	ND	0.02	**0.05/26.0 (0.001)**	0.10/1.1 (0.5)
Sydenham's chorea (*n *= 16)	**0.04/16.6 (0.003)**	**0.13/78.0 (0.0001)**	ND	0.12/1.5 (0.44)
Without RHD (*n *= 46)	0.04	0.04	**0.06/35.1 (0.002)**	0.08/1.0 (0.61)
Control subjects (*n *= 100)	0.004	0.002	0.002	0.09

Values are allele frequency/odds ratio (*p *value) and are reported only for significant associations (*p *< 0.05); *n *= number of haplotypes (for example, 140 alleles from 70 individuals and 200 alleles from 100 individuals). Entries in bold are statistically significant associations for patients versus controls. MVL, multivavular lesion (mitral regurgitation + aortic valve regurgitation); MVR, mitral valve regurgitation; ND, not determined; RF, rheumatic fever; RHD, rheumatic heart disease.

## Discussion

RHD is a sequela of group A β haemolytic streptococcal throat infection or scarlatine. M proteins are major targets of the host anti-streptococcal immune response [33-35]. Antigenic mimicry between streptococcal antigens, mainly M protein epitopes, and heart and brain components has been proposed as a triggering factor leading to autoimmunity in individuals with genetic predisposition [36,37].

HLA alleles regulate immune responses to infections, bind and present autoantigens with different affinities, play a role in T-cell repertoire selection, and may themselves be target autoantigens [5]. Several genetic markers of RHD susceptibility have been studied but no consistent association has been found [38-44]. However, associations with different HLA class II antigens have been observed amongst several population groups [12-17,21]. Since HLA class II antigens play an important role in antigen presentation to T-cell receptors, the variable association with HLA antigens is consistent with the possibility that different serotypes of group A streptococci could be implicated in RF and RHD in different countries.

Analyzing the role of HLA class II alleles/haplotypes in various diseases, it is important to consider that protective associations are equally, if not more, relevant than predisposing associations. Differential presentation of autoimmune peptides by protective and non-protective or susceptibility alleles can have major effects on the development of pathogenic autoimmunity. Future structure-function studies may reveal mechanisms by which certain alleles (for example, DQB1*0602) and the DRB1*06-13; 14-/DQA1*0102/DQB1*0602 haplotype may confer protection against RHD.

The heart is considered as an immunocompetent organ, and is consequently under immune surveillance by lymphocytes and macrophages. Dendritic cells expressing HLA class I and class II molecules at their surface and with the ability to present antigens to T lymphocytes have been described in the heart. In acute RF, Aschoff bodies (conglomerates of monocytes/macrophages and neutrophils) are frequently found in the heart and play an important role in the triggering of local inflammatory processes, acting as antigen-presenting cells. Superantigens are proteins derived from bacteria and viruses that polyclonally activate T cells by a MHC class II-dependent mechanism.

Autoreactivity to heart antigens caused by microbial infections has been described in several heart diseases [2,9,10,35,36,45-47]. The streptococcal M5 region comprises the immunodominant peptide M5, which is frequently recognized by peripheral T lymphocytes, especially in HLA DR7/DR53 severe RHD patients [4,35,36], suggesting that this peptide is preferentially presented to the T cell receptor in the context of HLA DR7/DR53 molecules and that it has a role, in combination with DQ molecules, in the development of severe valvular lesions [2-4,6]. Guilherme and colleagues [35] suggest that protein M1 is also significant in the development of RF because molecular mimicry occurs between M1, M5 and heart myosin and M6, M24 and brain proteins. Molecular mimicry between streptoccocal proteins and heart components has been proposed as the triggering factor of RHD, and CD4+ T cells have been found predominantly at pathological sites in the heart of RHD patients. The pathogenic mechanisms involved in the development of RF/RHD remain unclear. However, it is evident that an abnormal humoral and cellular immune response occurs.

Given the previously reported association of the HLA DRB1*07, DQB1*0302 and DQB1*0401-2 alleles with development of RF/RHD, we also assessed the ability of RHD-associated HLA genotypes to lead to the development of or to protect against RHD. Recently, it was shown that DRB1*0701 and DQA1*0201 are associated with mitral valve disease in Thailand, Turkey and the USA [5,18,48]. In our group of patients with RF, the DRB1*07/DQA1*0201 haplotype had a strong association in the Sydenhamn's chorea group and in the group of patients without RHD (Table 4). The RF risk allele DRB1*01 in combination with DQA1*0501 forms the risk genotype for the development of MVL, and DRB1*04 in combination with DQA1*0401 is the risk genotype for RF without RHD. In the present study in Latvia, the risk alleles appear to be DRB1*01 and DRB1*04, which is similar to results from Kudat and colleagues [8] and Wani and colleagues [14].

All RF patients in this study have the risk allele DQA1*0401 and the protective allele *0102, but the DOA1 risk genotypes are *0103/*0201 and *0301/*0501. There was no frequently found protective DQA1* genotype (Table 3). In the homogeneous patient groups, DQA1*0201, *0301 (in the Sydenhamn's chorea patient group), and *0401, *0501 and *0301 (in the MVL patient group) were frequently found as risk alleles (Tables 4 and 5). The risk allele DQA1*0201 was also found in homogeneous groups in other studies [5,18]. In the analysis of genotypes (Table 5), DQB1 risk alleles *0301-2 and *0401-2 together with the DQA1 allele *0501 were found relatively often in patients with MVL and together with the DQA1 allele *0301 in patients with Sydenhamn's chorea. Analyzing the RHD patient group, it is noticeable how frequently the DQA1 allele *0501 is found in the MVL patient group when in genotypes with DRB1*01 and DQB1*0301-2. The genotype DRB1/DQA1 *07/*0201, when in the haplotype with DQB1*0302, confers the risk of developing combined RHD-MVL (Table 6).

The DRB1*04/DQA1*0301/DQB1*0402-2 and DRB1*04/DQA1*0301/DQB1*0301 haplotypes are strongly associated with the Sydenhamn's chorea group (OR = 78.0, *p *< 0.0001 and OR = 16.6, *p *< 0.003, respectively; Table 6). It is interesting that the DRB1*15/DQA1*0102/DQB1*0602-8 haplotype was completely absent from all patient groups, whereas its frequency was 9% in control subjects (*p *< 0.61). Neither of these effects were significant (Table 6). However, the DQA1*0102/DQB1*0602-8 genotype found in this protective haplotype was individually associated with a protective effect in the control group (*p *< 0.00001; Table 5). The trend for a protective effect of this haplotype may have been conferred by the DQA1*0102 (OR = 0.34, *p *< 0.001; Table 1) and/or DQB1*0602-8 allele.

Interesting trends and associations are also clustered around the DRB1*06-related haplotypes. The DR6 antigen has two phenotypic splits encoded by the DRB1*13 or DRB1*14 alleles, each with several subtypes. In our previous study, the DRB1*06(13; 14) allele showed a significant protective effect against RF/RHD, and the DRB1*06(13; 14)/DQA1*0102 genotype was absent from all patient groups, which suggests that this haplotype has a protective influence. A negative association between RHD and DR6 was reported in the Utah study [38]. Therefore, depending on the DR6 splits (DR13 or DR14) or the DRB1*13/DRB1*14 suballeles and the nature of additional elements present in the same haplotype (that is, DQA and DQB alleles), the DR6 haplotypes may either confer risk for or protection against RHD.

It should be noted that the results from the Guedez and colleagues study [5] revealing that the DRB1*0701-, DR6-, and DQB1*0201-related haplotypes confer susceptibility to MVL are in agreement with those reported for RHD patients from USA, Turkish, Mexican, South African and Japanese populations, the majority of whom were in the MVL category [15,17,19,22,37,41,44].

While we can conclude that the development of severe combined RHD-MVL and Sydenhamn's chorea are caused by DQA1 alleles together with DRB1*07 and DRB1*04, respectively, the DRB1*07 allele in a haplotype with DQA1 risk alleles *0201 and DQB1*0302 leads to severe RF with MVL. DQB1*04 in a haplotype with DQA1*0401, 0301 and DQB1* risk alleles *0301, *0401-2 was discovered frequently in patients with Sydenham's chorea. Of course, as this study included only eight Sydenham's chorea patients, the group was too small for statistical significance, but we wished to show all the RF results.

According to results from other studies, ethnic differences are significant in genetics research. Guilherme and colleagues [6,9,33] have reviewed the ethnic differences in DR genotypes involved in predisposition for RF. In studies of DR07 [23] and haplotypes in Latvia (this study), DR01 and DR04 appear to be important. Data from DQ studies vary in different countries; for example, in Japan, patients with DQA1*0104 and DQB1*0503-1 develop severe RHD, which is different from Latvian data, while in the USA the DQA1*0201 allele and DRB1*0701/DQA1*0201 are common in RHD patients [38,41].

## Conclusion

Our findings indicate that the frequency of the HLA II class allele DQA1*0401 is significantly increased in DQA1 genotypes in RF patients compared to controls; alleles *0401, *0501,*0301 are increased in the RHD MVL group and *0201 is increased in the Sydenham's chorea group. The risk genotypes for RHD-MVL patients are DRB1*01/DQA1*0501 and DQA1*0501/DQB1*0301, and the risk haplotype is DRB1*07-/DQA1*0201-DQB1*0302. There are no valid data regarding the impact of DQA1 alleles on MVR patients in homogeneous patients groups.

Analysing genotypes and haplotypes, we can assume that DRB1*07 is responsible for a severe RF clinical outcome and, in a genotype with DQA1*0201/DQB1*0302, for severe combined RHD, which is similar to the conclusions of Guilherme and colleagues [6].

Risk genotypes for Sydenham's chorea patients are DRB1*07/DQA1*0201 and DQA1*0301/DQB1*0401-2, but the risk haplotypes are DRB1-/DQA1-DQB1 *04-/*0401-*0301 and *04-/*0301-*0401-2. There were no unambiguous allele findings in genotypes and haplotypes for Sydenham's chorea patients who also had MVL and MVR. Therefore, we consider that it is important to analyze RF patients in homogeneous patient groups.

Allele DQA1*0102 is protective for RF patients, but DQA1 and DRB1/DQA1 protective genotypes were not detected frequently. Genotype DQA1*0102/DQB1*0602 and haplotype DRB1*15-/DQA1*0102-DQB1*0602-8 can be assumed as protective for RHD.

The results of the present study support our hypothesis and indicate that certain HLA class II alleles, genotypes and haplotypes are associated with risk for or protection against RHD and that these associations are more evident in patients from clinically homogeneous groups. Also, ethnic differences should be taken into account in spite of the division in homogeneous groups, also presuming that, in the past five years, all studies have been performed with the PCR-SSP method.

Our study provides further information on the genetic predisposition for RF and the protective immune responses in RHD. Further insight into the molecular mechanisms of the disease will be a useful tool for predicting clinical outcome in RF patients and, thus, potentially offer new means and approaches to treatment and prophylaxis, including a potential vaccine.

## Abbreviations

AVR = aortal valve regurgitation; MVD = mitral valve disease; MVL = multivalvular lesion; MVR = mitral valve regurgitation; OR = odds ratio; PCR-SSP = PCR with amplification with sequence-specific primers; RF = rheumatic fever; RHD = rheumatic heart disease.

## Competing interests

The authors declare that they have no competing interests.

## Authors' contributions

SV made substantial contributions to the conception and design of the study, the acquisition, analysis and interpretation of data, was involved in drafting the manuscript and revising it critically for important intellectual content, and gave final approval of the version to be published. EJ analysed and interpreted data. ZD made contributions to the conception and design of the study, was involved in drafting the manuscript and revising it critically for important intellectual content, and gave final approval of the version to be published. SA made substantial contributions to the conception and design of the study, acquisition of data, and performed analysis and interpretation of data. ShR performed data acquisition. GD made contributions to the conception and design of the study and was involved in revising it critically for important intellectual content.
